# COVID-19 Vaccines Cost-Effectiveness Analysis: A Scenario for Iran

**DOI:** 10.3390/vaccines10010037

**Published:** 2021-12-29

**Authors:** Atefeh Vaezi, Alipasha Meysamie

**Affiliations:** 1 Applied Physiology Research Center, Cardiovascular Research Institute, Isfahan University of Medical Sciences, Isfahan 8174673461, Iran; a.vaezi@med.mui.ac.ir; 2 Department of Community Medicine, School of Medicine, Tehran University of Medical Sciences, Tehran 1416753955, Iran; 3 Community Based Participatory Research Center, Iranian Institute for Reduction of High Risk Behaviors, Tehran University of Medical Sciences, Tehran 1416753955, Iran

**Keywords:** SARS-CoV-2, effectiveness, SARS-CoV-2 vaccines, cost-effectiveness analysis

## Abstract

COVID-19 vaccines are supposed to be critical measure for ending the pandemic. Governments had to decide on the type of vaccine to provide for their population. In this decision-making process, cost-effectiveness analysis is considered a helpful tool. This study is a cost-effectiveness analysis utilized to calculate the incremental cost per averted disability-adjusted life year (DALY) by vaccination compared to no vaccination for different COVID-19 vaccines. The incremental cost-effectiveness ratio (ICER) for a vaccination with COVID-19 vaccines was estimated at 6.2 to 121.2 USD to avert one DALY and 566.8 to 10,957.7 USD per one death. The lowest and highest ICERs belong to Ad26.COV2.S and CoronaVac, respectively. Considering the scenario of Iran, vaccines that are recommended include ad26.cov2.s, chadox1-S, rAd26-S + rAd5-S, and BNT162b2 in the order of recommendation.

## 1. Introduction

At the beginning of 2020, the world encountered a new disease, which was later named the coronavirus disease 2019 (COVID-19). On 7 January, the causative agent was isolated, and gene sequences were revealed [1]. COVID-19, an atypical pneumonia caused by SARS-CoV-2, spread fast globally and was declared as a pandemic on 11 March 2020 [2]. By the end of September 2021, more than 233 million cases, with about 4.7 million deaths, were reported worldwide [3]. In Iran, the first definite COVID-19 case was reported on 19 February 2020, and the virus spread fast all over the country. Recent reports show 5.5 million coronavirus cases and more than 119,000 deaths by 28 September 2021 [4].

Public health measures for the prevention of COVID-19 include wearing masks, hand washing, keeping physical distance, and coughing or sneezing etiquette [5]. Due to the incredible spread of the virus and the risk of transmission from asymptomatic carriers [6], vaccination is assumed to be the most effective public health measure to prevent COVID-19 and to control the burden of disease due to severe cases and deaths [7,8].

COVID-19 is a human-to-human transmitted disease that is transmitted via droplets and airborne particles [9]. Studies report the basic reproduction number of SARS-CoV-2 from 1.4 to 7.23 [10]. The causative agent of COVID-19 is an enveloped RNA virus from the coronavirus family. There is a structural spike (S) protein on the virus envelope, which is essential for the entrance of the virus to the host cell. Most COVID-19 vaccines are focused on the S protein, which is expressed as a recombinant protein or delivered via viral vectors or as DNA or mRNA vaccines [11].

According to the vaccine landscape by the World Health Organization (WHO) [12], as of 27 August 2021, 112 vaccines are in clinical development. Eight vaccines are in phase 4, including CoronaVac (by Sinovac Biotech Research and Development Company, Beijing, China), BBIBP-CorV (by Sinopharm, China National Pharmaceutical Group Corporation (CNPGC), Beijing, China), ChAdOx1-S (by AstraZeneca, Cambridge, England and University of Oxford), recombinant novel coronavirus vaccine (adenovirus type 5 vector) (by Cansino biological Incorporated, Tianjin, China), Ad26.COV2.S (by Janssen Pharmaceutical, Beerse, Belgium), mRNA-1273 and mRNA-1273.351 (by Moderna, Cambridge, Massachusetts, USA and National Institute of Allergy and Infectious Diseases), and BNT162b2 (by Pfizer/BioNTech, Manhattan, New York, NY, USA- Mainz, Germany); and 21 vaccines are in phase 3 of the study (Table 1). On 11 December and 18 December 2020, the Food and Drug Administration (FDA) announced the first and second Emergency Use Authorization (EUA) for BNT162b2 and mRNA-1273 vaccines, respectively. About two months later, Ad26.COV2.S obtained the third EUA from FDA [13]. FDA approved BNT162b2 on 23 August 2021 for the prevention of COVID-19 disease in 16 years and older individuals [14].

Based on published reports, it seems that vaccines are generally successful in preventing severe disease and death, as well as preventing asymptomatic infection in fully vaccinated individuals; vaccines are almost well-tolerated, and reports on serious complications are rare [15]. Today, after about ten months of introducing the first COVID-19 vaccine, more than 6.13 billion doses have been administered worldwide. The percentage of the fully vaccinated population varies widely from 84% in Malta, United Arab Emirates, and Portugal to less than 1% in some other countries. Over 43 million vaccine doses have been administered in Iran, and about 17% of the population is fully vaccinated so far in using BBIBP-CorV, BBV152, rAd26-S + rAd5-S, ChAdOx1-S, and COVIran Barekat vaccines [16].

Similarly to any other virus, SARS-Cov-2 had mutations in its genetic sequence. These mutations may increase infectivity, severity, and the transmission rate of the virus and decrease the effectiveness of public health measures. They may pose an increased risk to global public health [17,18]. From the Variants of Concern (VOCs), the Alpha variant (B.1.1.7) was firstly documented in September 2020 in the United Kingdom; this variant has a mutation in the spike protein and increases the viral load of SARS-Cov-2 in the upper respiratory tract [19]. The next VOC is the Beta variant, also known as B.1.351. This variant was documented in South Africa in May 2020 and designated on 18 December 2020; the mutation causes higher transmissibility and immune escape [20]. Other VOCs include Gamma (P.1) and Delta (B.1.617.2) variants. The Gamma variant, which was documented in Brazil in November 2020, can escape both natural and vaccine-induced immunity, with a higher rate of severe infection and mortality [21]. The Delta variant was documented in India in October 2020 and designated on 4 April 2021; this variant caused the resurgence of COVID-19 in India and is more transmissible than the Alpha variant [22]. Data on the effectiveness of vaccines against each variant are scarce. A recent study on two COVID-19 vaccines (BNT162b2 and ChAdOx1-S) shows a slight decrease in the effectiveness of these two vaccines against the Delta variant compared to the Alpha variant. This difference was notable for recipients with one dose, suggesting the need for complete vaccination [23]. However, there are some concerns about administering a booster dose considering the waning of vaccines’ effectiveness over time [24].

Although reaching herd immunity for COVID-19 is not imaginable without vaccination, one crucial aspect in deciding for vaccination is the financial and sustainability aspects. Moreover, due to the scarce resources, countries need to develop country-specific vaccination programs considering the type of vaccine and the priorities. In this regard, cost-effectiveness analysis in the context of health technology assessments is helpful [25]. In this study, we conducted a cost-effectiveness analysis for COVID-19 different vaccines in order to determine the best choice with sufficient impact for the country or at least showing the cost of saving a life by investing in vaccines. This study can provide evidence for policymakers and may help them in deciding on the type of vaccine with priority for the country.

## 2. Material and Methods

This cost-effectiveness analysis was carried to compare the impact of COVID-19 vaccines on averted death and DALY in Iran. Vaccines that had published the result of their phase 3 trial were included in this study. The WHO COVID-19 vaccine dashboard was reviewed, and the reports of phase 3 trials for each vaccine were extracted by searching PubMed.

### 2.1. The Cost-Effectiveness Model/Measurements

#### 2.1.1. Demographic Data

Data regarding the population of Iran were obtained from the Statistical Center of Iran based on the 2016 population survey [26].

#### 2.1.2. Disease Burden Estimation

The incidence, prevalence, and mortality of COVID-19 were obtained from WHO reports [3]. The severity of the disease is assumed as mild to moderate (80% of cases), severe (15% of cases), and critical disease (5% of cases). Mild to moderate cases include symptomatic individuals without hypoxia or with SpO2 at more than 90% in room air and who do not need hospitalization. Severe COVID-19 is defined as cases with SpO2 less than 90% and severe respiratory distress that requires hospitalization and oxygen support. Critical disease is defined as those with acute respiratory disease syndrome who require critical care [27].

Data regarding the DALY were obtained from a study by Mohanty et al., which reports the impact of COVID-19 on DALY in the USA, Italy, Sweden, and Germany [28]. Another study was also used, which documents the impact of COVID-19 on the life expectancy of the US population [29].

#### 2.1.3. Cost Measurements

The cost of hospitalization of COVID-19 patients in Iran was obtained from the study by Ghaffari et al., which estimated the direct and indirect cost of treatment of COVID-19 patients (29) and interviews of the general manager of the social security insurance organization of Iran [30]. The reported cost per dose for each vaccine varies widely and was obtained from the news [31,32,33,34]. As the final cost per dose is defined in contracts between governments and companies and may differ based on different issues, we used the highest reported price a country paid to ensure that the cost is not underestimated.

#### 2.1.4. Effectiveness Measurements

The efficacy of all vaccines except CoronaVac was obtained from the published data of vaccines’ phase III trials. Reports of news agencies were used for the CoronaVac vaccine’s efficacy. The effectiveness of all vaccines except rAd26-S + rAd5-Svwas obtained from effectiveness studies. The effectiveness of the rAd26-S + rAd5-S vaccine was calculated using the reduction rate in the efficacy of the ChAdOx1-S vaccine based on effectiveness studies.

The herd immunity level was set at 50% and 75%; the percentage of the vaccinated population needed in order to reach herd immunity was calculated by multiplying efficacy/ effectiveness by herd immunity level.

We run four different scenarios based on additional data of DALY, different herd immunity levels, and by considering the administration of a booster dose. In Scenario 1, which is the base scenario, we assumed DALY as 1.13 [29]; the level of herd immunity was set at 50%, with basic doses of vaccines (two doses for all vaccines except for Ad26.COV2.S, which is a single dose vaccine). In Scenario 2, we changed DALY to 4 [28]; other measures were not changed. In Scenario 3, we considered adding a booster dose, with herd immunity levels of 50% and DALY of 1.13. In Scenario 4, we assumed herd immunity level at 75%, DALY of 1.13 and adding a booster dose. Considering emerging virus mutations, waning vaccine immunity in different countries, the similarity of COVID-19 pandemic to previous influenza pandemic which resulted in recommended annual vaccination, and global debates on adding a booster dose, we assume adding a booster dose for all vaccines in our 3rd and 4th scenarios.

## 3. Results

From 29 vaccines in their phase 3 and 4 trials, seven vaccines with reported efficacy/ effectiveness in a published article or the news were included in this study. Table 2 presents and summarizes the different characteristics of vaccines included in the cost-effectiveness analysis.

### 3.1. Vaccines Profile

**CoronaVac** was developed by Sinovac Research and Development Company in China. The platform of this vaccine is an inactivated virus: two dose vaccine scheduled on days 0 and 14, intramuscular (IM) [12]. The phase 1/2 clinical trial (registered number: NCT04352608) was published in November 2020. Participants numbering 143 (in phase I) and 600 (in phase II) received either three micrograms, six micrograms, or a placebo on days 0–14 or 0–28. The rate of adverse events was lower in the three-microgram dose group. The rate of seroconversion of neutralizing antibodies on day 28 was higher in the schedule of 0–28 in comparison to 0–14. The most commonly reported adverse event was pain at the injection site [35]. Based on the reports by Bloomberg, the efficacy of CoronaVac was 78% in Brazil [36], 91.25% in Turkey [37], and 65% in Indonesia [38]. In a study on the effectiveness of CoronaVac in Brazil, this vaccine was more effective against death (61.2, 95%CI: 48.9-70.5) and hospital admission (55.5%, 95% CI: 46.5–62.9) in comparison to symptomatic infection (46.8%, 95% CI: 38.7–53.8) [39].

**BBIBP-CorV** was developed by the Beijing Institute of biological products/Sinopharm and was authorized for emergency use by WHO. The vaccine platform is an inactivated virus, scheduled at days 0 and 21 via the IM route [12]. The phase I study results on 192 participants and phase II on 448 participants show that BBIBP-CorV is well tolerated with fever as the most reported adverse event; the four microgram vaccine induced a higher titer of neutralizing antibody [40]. According to the report of WHO, the overall efficacy, the efficacy against hospitalization, and the effectiveness of this vaccine are 78.1% (95% CI: 64.9–86.3), 78.7% (95% CI: 26.0–93.9), and 90% (95% CI: 88–91), respectively [41].

**ChAdOx1-S** is a COVID-19 vaccine developed by the University of Oxford and AstraZeneca Company. This is a chimpanzee adenovirus vectored vaccine planned in two doses of IM injection on a 0–28 schedule [12]. In phase I/II clinical trial, 1077 healthy adults aged 18–55 years were enrolled. Neutralizing antibodies were detected in all participants after the second dose. Reported adverse events include pain, fever, muscle ache, headache, chills, and malaise, all of which were categorized as mild [42]. The first report of the phase 3 trial was published in December 2020; this was an interim analysis of four randomized controlled trials in the United Kingdom, South Africa, and Brazil. In the efficacy analysis, 11,636 participants were included. Vaccine efficacy in participants who received two standard doses was 62.1% (95% CI: 41.0–75.7); in participants who received a low dose followed by the standard dose, it was 90.0% (95% CI: 67.4–97.0) [43]. In another study, vaccine efficacy after a single dose was 76% without waning of the protection for three months; moreover, the efficacy in the schedule of 0–90 days was 82.4% (95% CI: 62.7–91.7) [44]. The effectiveness of ChAdOx1-S was calculated in a study against alpha and delta variants considering one and two doses. The effectiveness against Alpha variant after one and two doses was 48.7% (95% CI: 45.2–51.9) and 74.5% (95% CI: 68.4–79.4), respectively. Considering the Delta variant, the effectiveness of this vaccine decreased to 30.0% (95% CI: 24.3–35.3) and 67.0% (95% CI: 61.3–71.8) in one dose recipients and fullly vaccinated individuals, respectively [23].

**rAd26-S + rAd5-S** is an adenovirus vectored COVID-19 vaccine and was developed by Gamaleya Research Institute and funded by the Health Ministry of the Russian Federation. This is a two doses vaccine with a schedule of 0–21 days and IM injection. Human adenovirus vector 26 was used for the first vaccine, and human adenovirus vector five was used for the second vaccine [12]. The result of phase I on either type of vaccine shows safety without any serious adverse events. Based on the phase II trial results, in which 20 participants received rAd-26 followed by rAd-5, the seroconversion rate was 100% [45]. The phase III study on 19866 participants reports the efficacy of 91.6% (95% CI: 85.6–95.2). The vaccine’s efficacy in prevention against moderate and severe diseases was 100% (95% CI: 94.4–100) [45]. The Gamaleya institute announced a 97.6% effectiveness for this vaccine based on the outcomes of 3.8 million Russians vaccinated with two doses in April 2021 [46].

**mRNA-1273** is an RNA-based vaccine developed by Moderna Company and the National Institute of Allergy and Infectious Disease (NIAID). It is scheduled on days 0–28 via IM injection [12]. The vaccine should be stored at −20 °C. It could be stored at 2–8 °C for one month. Based on a phase I trial report on 45 healthy 18–55 year old participants, adverse events include fatigue, chills, headache, myalgia, and pain at the injection site. Three participants reported severe adverse events after the administration of the second dose. Seroconversion was reported in 100% of the participants [47]. The other phase I study on older adults (40 healthy 56 years and older) demonstrates a 100% immune response. Adverse events were mostly mild and moderate, including fatigue, chills, headache, myalgia, and pain at the injection site [48]. Based on the phase III trial report, which included 30,420 participants, the vaccine’s efficacy was 94.1% (95% CI: 89.3–96.8) in prevention against COVID-19 illness [49]. The effectiveness of mRNA-1273 against symptomatic COVID-19 infection 14 days after the first dose was 72% (95% CI; 62–80), and seven days after the second dose it was 94% (95% CI: 86–97); the effectiveness of this vaccine against severe outcomes, e.g., hospitalization or death in fully vaccinated individuals was 96% (95% CI: 74–100) [50].

**BNT162b2** is an RNA-based COVID-19 vaccine developed by Pfizer/BioNTech and Fosun Pharma with a schedule of two doses on days 0–21 via the IM route [12]. The vaccine should be stored in minus 70° centigrade for a long term, and it could be stored in 2–8° centigrade for five days. The phase I/II study on 45 healthy adults (18–55 years old) supports the induction of neutralizing antibodies. Pain at the injection site was reported by most of the participants. Other reported adverse events include fatigue, headache, chills, muscle pain, and joint pain [51]. The efficacy of the vaccine reported by a phase III trial on 43,548 participants (16 years and older) was 95% (95% CI: 90.3–97.6) [52]. The effectiveness of BNT162b2 was estimated in a test-negative design study as 91% (95% CI: 88–93) against symptomatic disease, which rises to 96% (95% CI: 82–99) against severe outcomes in a fully vaccinated population [50].

**Ad26.Cov2.S** is a non-replicating viral vector COVID-19 vaccine manufactured by the Janssen Pharmaceutical company. The suggested schedule for this vaccine is a single dose schedule or a 56 days apart two doses [12]. In a phase-I study on 25 participants, one or two injections were administered. By day 57, after the first dose, all participants showed binding and neutralizing antibodies [53]. A phase 3 trial on 39,321 participants shows the efficacy of 66.5% (95% CI: 55.5–75.1) against symptomatic COVID-19 infection [54]. The effectiveness of the vaccine against hospitalization was 84% (95% CI: 64–93) and 85% (95% CI: 72–92) in 65–74 and over 75 year old adults, respectively [55].

**CovIran Barekat** is an inactivated whole virus vaccine against Sars-Cov-2 manufactured by the Shifapharmed Industrial Group Company in Iran. The animal trial result, published as a preprint, shows that this vaccine can induce an immune response with adequate safety in mice, rabbits, and non-human primates [56]. Study protocols were registered in the Iranian Registry of Clinical trials (IRCT20201202049567N1, IRCT20201202049567N2, and IRCT20201202049567N3). The results of the trials are not published yet but were announced in the press. In phase II of the study, 12 cases of COVID-19 (3/ 56 cases in placebo and 9/ 224 in vaccine group) were reported [57]. This vaccine is widely used in Iran, but as there is no published report on its efficacy or effectiveness by the date of drafting this study; thus, we exclude this vaccine from cost-effectiveness analysis.

### 3.2. Cost-Effectiveness

We used COVID-19 vaccines’ efficacy and effectiveness and various treatment and vaccine cost measures to estimate the Incremental Cost-Effectiveness Ratio (ICER) of vaccination compared to no vaccination.

In the absence of any COVID-19 vaccine in Iran (population, 79,926,270), the infection rate was 17.6 per 1000 (1,411,731 cases), and about 57,889 deaths were reported [3]. The average hospitalization and ICU admission rates were 42.2% and 15%, respectively [58].

In the base case scenario, based on the reported vaccines’ effectiveness, the Ad26.Cov2.S, which is a single dose vaccine, has the lowest ICER for death and DALY (566.8 and 6.2 USD, respectively). The highest ICER belongs to CoronaVac (10,957.7 for death and 121.2 for DALY). The cost benefit for investing in vaccine is 105.6 for Ad26.Cov2.S, which is the highest, and 8.9 for Coronavac, which is the lowest benefit.

In the second scenario, we changed the value of DALY from 1.13 (in the base scenario) to 4. The ICER for DALY decreases for all vaccines by almost 3.5-fold. Still, the lowest and highest ICER for DALY belongs to Ad26.Cov2.S (1.7 USD) and CoronaVac (34.2 USD), respectively.

Considering a booster dose in the third scenario (keeping other measures the same as the base scenario), the lowest ICER for DALY belongs to Chadox1-S and Ad26.Cov2.s, which is 12.5 USD. The ICER for death is almost increased compared to the base scenario. The highest ICER for death belongs to Coronavc (16,436 USD), and the lowest belongs to Ad26.Cov2.S (11.33 USD). Considering the cost-benefit measure, by investing one USD in ChAdOx-1-S, the government would save 59.0 USD. The lowest benefit would be obtained by investing in CoronaVac, which is 5.6 USD for one USD.

**Figure 1:**
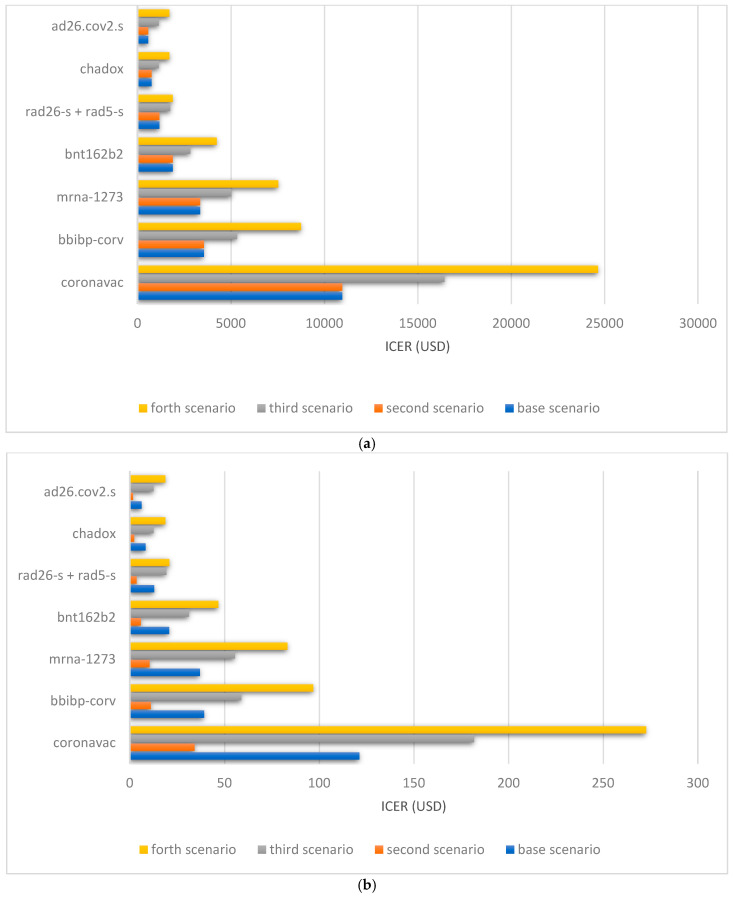
(**a**) ICER for the prevention of death of COVID-19 in cost-effectiveness analysis considering Iran’s situations. (**b**) ICER for the prevention of DALY of COVID-19 in cost-effectiveness analysis considering Iran’s situations. In base scenario, the DALY was assumed 1.13 with herd immunity level of 50% and basic doses of vaccines; in Scenario 2, we assumed DALY as 4. In Scenario 3, we considered adding a booster dose; in Scenario 4, herd immunity level was set at 75% and, a booster dose added. Abbreviations: DALY, disability adjusted life year; ICER: incremental cost-effectiveness Ratio; USD: United States Dollar.

In the fourth scenario, the herd immunity level is set at 75% with consideration of a booster dose. The ICER for DALY using a Chadox1-S or Ad26.Cov2.S would be 18.8 USD. The cost-benefit for Chadox1-S would be 36.9, and for Ad26.Cov2.S it would be 32.6. Figure 1 demonstrates the ICER for all vaccines by different assumptions.

The detailed results of cost-effectiveness analysis for different vaccines are demonstrated in Table 3.

## 4. Discussion

With the emergence of COVID-19, governments had to implement public health measures in order to protect their population. By introducing COVID-19 vaccines, studies and reports demonstrate the overall effectiveness of vaccines against COVID-19 [59,60]. The decision on the type of vaccine to be administered could be another challenge for the governments. In this process, cost-effectiveness analysis studies are beneficial and mentioned as a decision support tool. This analysis provides the policymakers with information on whether the health gain associated with new technology (such as a vaccine) is worth the invested cost. Due to the fact that cost-effectiveness analysis is influenced by vaccine efficacy/ effectiveness, disease burden, population variables, vaccine price, and treatment cost, the results could be localized and may change based on countries’ situations [61].

This study is a cost-effectiveness study on the administration of COVID-19 vaccines compared to no vaccine, considering the situation in Iran. By drafting this manuscript, five different COVID-19 vaccines are introduced in Iran, including Sinopharm, Bharat, AstraZeneca, Sputnik, and CovIran Barekat.

It should be noted that the suggested number of doses for all vaccines is two doses, except for Ad26.Cov2.S, which is a single-dose vaccine. The cost of preventing one death using CoronaVac is 10957 USD in the base scenario, which is the highest cost. If the government administers Ad26.Cov2.S, this would cost about 566 USD/death. The second lowest cost for the prevention of one death belongs to ChAdox1-S, which is 756.7 USD. The ICER for preventing one death using BBIBP-CorV and rAd26-S + rAd5-S, two widely administered vaccines in Iran, is 3555.5 USD and 1166 USD, respectively. Considering the reported vaccines’ effectiveness in the base case scenario, the ICER for every DALY averted differs from 6.2 to 121.2. Other vaccines’ ICER is somewhere in between. The increases in the number of deaths caused by COVID-19 in the absence of vaccination would result in an increase in DALY [28]. The difference between the base case scenario and second scenario shows that by an increase in DALY, the cost for averting one DALY would decrease.

Considering the threshold for willingness to pay at GDP per capita, which was 2282 USD [62] in 2020 for Iran, all vaccines are highly cost-effective in the aversion of DALY (cost per DALY less than GDP per capita). This would be different for the prevention of death. The administration of CoronaVac is not cost-effective (cost per death more than 3GDP per capita) with 10,957 USD/death. Investments in BBIBP-CorV and mRNA-1273 are cost-effective (cost per death between 1-3GDP per capita); others are highly cost-effective. The result of cost-effectiveness analysis in Turkey demonstrates that any vaccine with an effectiveness of more than 80% and coverage of more than 30% would be cost saving. With coverage of about 50%, the vaccine’s effectiveness should be at least around 55% to be cost-saving [63]. In another study in South Africa, it has been shown that coverage of 40% of the population, which would prevent more than 73,000 deaths. It also shows that vaccination would be cost saving with a cost of up to 25 USD/person and vaccine effectiveness of at least 20%, 30%, and 40% against infection, symptomatic, and critical disease, respectively [64].

Considering the cost benefit index, for every one dollar investment in vaccine provision, we would save between 8.9 USD (for CoronaVac) to 105.6 USD (for Ad26.COV2.S). Vaccination with BNT162b2 (effectiveness of 91%), rAd26-s + rAd5-s (effectiveness of 82%), and ChAdOx1-S (effectiveness of 74.5%) would save 28.6, 51.5, and 89.1 USD, respectively. Adding a booster dose would decrease the benefit, which is obvious when comparing the base scenario with the third scenario, but still remains cost beneficial. In the third scenario, the cost benefit for administering Ad26.COV2.S and ChAdOx1-S would be 52.3 and 59.0, respectively; moreover, the lowest benefit is be obtained by investing in CoronaVac, which would be 5.6.

We tried to use the best-reported estimates for different measures. Still, unpublished data with respect to some vaccines and the costs of treatments could be mentioned as a limitation of this study. Furthermore, we did not add the costs of distribution, injection, and maintenance of the vaccine in the field. Considering the different maintenance conditions for various vaccines, this could be considered as another limitation.

## 5. Conclusions

In this study, four different scenarios were assumed, and all assumptions show that investing in COVID-19 vaccine would be cost effective considering DALY. Considering death, some vaccines are highly cost effective, including Ad26.Cov2.S, ChAdOx1-S, and rAd26-S + rAd5-S in all scenarios. From the vaccines that are available in Iran, ChAdOx1-S is a highly cost-effective vaccine and has a cost-benefit of 89.1. The rAd26-S + rAd5-S in the second place with ICER of 1166 USD and cost-benefit of 51.5 is another highly cost-effective COVID-19 vaccine for Iran. BBIBP-CorV would save 8.9 USD for one USD with 3555.5 USD for the prevention of every death and is a cost-effective vaccine. Altogether, considering ICER/DALY, ICER/death, cost-benefit, and sufficiency of data, the recommended vaccines for Iran include Ad26.Cov2.S, ChAdOx1-S, rAd26-S + rAd5-S, and BNT162b2.

## Figures and Tables

**Table 1:** Basic information on COVID-19 Vaccines in phases 3 and 4 [12].

	Phase of Study	Developers	Vaccine Name	Vaccine Platform	Route of Administration	Number of Doses/Schedule
1	4	Sinovac Research and Development Co., Ltd.	CoronaVac	Inactivated virus	IM	2/day 0 + 14
2		Sinopharm + China national Biotech group Co + Beijing Institute of Biological Products	BBIBP-CorV	Inactivated virus	IM	2/day 0 + 21
3		AstraZeneca + University of Oxford	ChAdOx1-S-(AZD1222)	Viral vector (Non-replicating)	IM	1–2/day 0 + 28
4		CanSino biological Inc./Beijing institute of biological products	Recombinant novel coronavirus vaccine (adenovirus type 5 vector)	Viral vector (non-replicating)	IM	1/day 0
5		Janssen Pharmaceutical	Ad26.COV2.S	Viral vector (non-replicating)	IM	1–2/day 0 or day 0 + 56
6		Moderna + National Institute of Allergy and Infectious Diseases	mRNA -1273	RNA based vaccine	IM	2/day 0 + 28
7		Moderna + National Institute of Allergy and Infectious Diseases	mRNA-1273.351	RNA based vaccine	IM	3/day 0 or day 0 + 28 or day 56
8		Pfizer/BioNTech + Fosun Pharma	BNT162b2 (3 LNP-mRNAs), also known as “Comirnaty”	RNA based vaccine	IM	2/day 0 + 21
9	3	Sinopharm + China National biotech Group Co + Wuhan Institute of Biological Products	Inactivated SARS-CoV-2 vaccine (Vero cell)	Inactivated virus	IM	2/day 0 + 21
10		Gamaleya Research Institute; Health Ministry of the Russian Federation	Gam-COVID-Vac Adeno-based (rAd26-S + rAd5-S)	Viral vector (Non-replicating)	IM	2/day 0 + 21
11		Novavax Gaithersburg, Maryland, USA	Sars-cov-2 rS/Matrix M1-adjuvant (full length recombinant SARS cov-2 glycoprotein nanoparticle vaccine adjuvant with Matrix M) NVX-CoV2373	Protein subunit	IM	2/day 0 + 21
12		Anhui Zhifei Longcom Biopharmaceutical + Institute of Microbiology, Chinese Academy of Sciences	Recombinant SARS-CoV-2 vaccine (CHO Cell)	Protein subunit	IM	2–3/ Day 0 + 28 or Day 0 + 28 + 56
13		CureVac AG Tübingen, Germany	CVnCoV Vaccine	RNA based vaccine	IM	2/ Day 0 + 28
14		Institute of Medical Biology + Chinese Academy of Medical Sciences	SARS-CoV-2 vaccine (Vero cells)	Inactivated virus	IM	2/ Day 0 + 28
15		Research Institute for Biological Safety Problems, Rep. of Kazakhstan	QazCovid-in^®^-COVID-19 inactivated vaccine	Inactivated virus	IM	2/ Day 0 + 21
16		Zydus Cadila Ahmedabad, Gujarat, India	nCov vaccine	DNA based vaccine	ID	3/ Day 0 + 28 + 56
17		Bharat Biotech International Limited, Hyderabad, India	Whole-Virion Inactivated SARS-CoV-2 Vaccine (BBV152): covaxin	Inactivated virus	IM	2/ Day 0 + 14
18		Sanofi Pasteur + GSK Lyon, France-London, England	VAT00002: SARS-CoV-2 S protein with adjuvant	Protein subunit	IM	2/ Day 0 + 21
19		Shenzhen Kangtai Biological Products Co., Ltd. Shenzhen, China	Inactivated sars-cov-2 vaccine (verocell)	Inactivated virus	IM	2/day 0 + 28
20		Vaxine Pty Ltd./CinnaGen Co Adelaide, Australia-Tehran, Iran	COVAX-19	Protein subunit	IM	2/day 0 + 21
21		Instituto Finlay de Vacunas Havana, Cuba	FINLAY-FR-2 anti-SARS-CoV-2 Vaccine (RBD chemically conjugated to tetanus toxoid plus adjuvant)	Protein subunit	IM	2/Day 0 + 28
22		Federal Budgetary Research Institution State Research Center of Virology and Biotechnology “Vector” Koltsovo, Novosibirsk Oblast, Russia	EpiVacCorona (based on peptide antigens for the prevention of COVID-19)	Protein subunit	IM	2/Day 0 + 21
23		West China Hospital + Sichuan University	RBD (baculovirus production expressed in Sf9 cells) recombinant SARS-CoV-2 vaccine (Sf9 Cell)	Protein subunit	IM	2/day 0 + 28
24		Academy of Military Science (AMS), Walvax biotechnology, and Suzhou Abogen Biosciences Shanghai, China	SARS-cov-2 mRNA vaccine (ARCoV)	RNA based vaccine	IM	2/day 0 + 14 or day 0 + 28
25		Center for Genetic Engineering and Biotechnology (CIGB) Havana, Cuba	CIGB-66 (RBD+aluminium hydroxide)	Protein subunit	IM	3/Day 0 + 14 + 28 or Day 0 +28 + 56
26		Valneva, National Institute for Health Research, United Kingdom	VLA2001	Inactivated virus	IM	2/day 0 + 21
27		Nanogen pharmaceutical biotechnology Ho Chi Minh, Vietnam	Recombinant sars-cov-2 spike protein, aluminum adjuvant (nanocovax)	Protein subunit	IM	2/day 0 + 21
28		Erciyes University, Turkey	ERUCOV-VAC, inactivated virus	Inactivated virus	IM	2/day 0 + 21
29		SK Bioscience Co. Gyeonggi-do, South Korea	GBP510	Protein subunit	IM	2/day 0 + 28

Abbreviation: DNA: deoxyribonucleic acid; ID: intra dermal; IM: intra muscular; RNA: ribonucleic acid; SARS-CoV-2: severe acute respiratory syndrome coronavirus 2.

**Table 2:** Demographic data on COVID-19 vaccines in cost-effectiveness analysis.

Developers	Vaccine Name	Country	Type of Candidate Vaccine	Number of Doses/Route	Timing of Doses (Days)	Price per Dose
Sinovac Research and Development Co.	CoronaVac	China	inactivated	2/IM	0, 14	30 USD
Beijing Institute of Biological Products/Sinopharm	BBIBP-CorV	China	inactivated	2/IM	0, 21	36 USD
Moderna/NAIAD	mRNA-1273	USA	LNP-encapsulated mRNA	2/IM	0, 28	37 USD
BioNTech/Fosun Pharma/Pfizer	BNT162b2	USA, Germany	3 LNP-mRNAs	2/IM	0, 21	19.5 USD
Gamaleya Research institute/ Sputnik	rAd26-S+rAd5-S	Russia	Adenovirus base	2/IM	0, 21	10 USD
University of Oxford/ AstraZeneca	ChAdOx1-S	UK	chimpanzee adenovirus-vectored vaccine	2/IM	0, 28	5.5 USD
Janssen Pharmaceutical	Ad26.COV2.S	USA, Netherland	Viral vector (non-replicating)	1–2/IM	0 or 0, 56	10 USD

Abbreviation: IM: intra muscular; RNA: ribonucleic acid; NAIAD: National Institute of Allergy and Infectious Disease; UK: United Kingdom; USA: United States of America; USD: United States Dollar.

**Table 3:** Different cost/effectiveness scenarios considering different COVID-19 vaccines.

		Base Scenario			Scenario 2			Scenario 3			Scenario 4		
Vaccine Name		ICER Death	ICER DALY	Cost-Benefit	ICER Death	ICER DALY	Cost-Benefit	ICER Death	ICER DALY	Cost-Benefit	ICER Death	ICER DALY	Cost-Benefit
CoronaVac	Efficacy: 0.78	3944.7	43.6	15.5	3944.7	12.3	15.5	5917.1	65.4	10.0	8875.7	98.1	5.9
	Effectiveness: 0.46	10,957.7	121.2	8.9	10,957.7	34.2	8.9	16,436.5	181.8	5.6	24,654.8	272.7	3.1
BBIBP-CorV	Efficacy: 0.78	4721.6	52.2	12.7	4721.6	14.7	12.7	7082.4	78.3	8.1	10,382.9	114.8	4.8
	Effectiveness: 0.9	3555.5	39.3	14.8	3555.5	11.1	14.8	5333.3	58.9	9.5	8761.4	96.9	5.3
mRNA-1273	Efficacy: 0.94	3342.8	36.9	15.1	3342.8	10.4	15.1	5014.2	55.4	9.7	7521.3	83.2	5.7
	Effectiveness: 0.94	3349.9	37.0	15.1	3349.9	10.4	15.1	5024.8	55.5	9.7	7537.3	83.3	5.7
BNT162b2	Efficacy: 0.95	1728.5	19.1	29.9	1728.5	5.4	29.9	2592.7	28.6	19.6	3889.1	43.0	12.0
	Effectiveness: 0.91	1883.8	20.8	28.6	1883.8	5.8	28.6	2825.7	31.2	18.7	4238.6	46.8	11.7
rAd26-S + rAd5-s	Efficacy: 0.91	953.4	10.5	57.1	953.4	2.9	57.1	1430.1	15.8	37.7	2145.2	23.7	23.4
	Effectiveness: 0.82	1166.8	12.9	51.5	1166.8	3.6	51.5	1750.3	19.3	34.0	1889.6	20.9	25.0
ChAdOx1 nCoV-19	Efficacy: 0.82	618.5	6.8	98.6	618.5	1.9	98.6	927.8	10.2	65.4	1391.8	15.3	40.9
	Effectiveness: 0.74	756.7	8.3	89.1	756.7	2.3	89.1	1135.0	12.5	59.0	1702.6	18.8	36.9
Ad26.COV2.S	Efficacy: 0.66	904.5	10.0	83.4	904.5	2.8	83.4	1809.0	20.0	41.2	2713.5	30.0	25.6
	Effectiveness: 0.84	566.8	6.2	105.6	566.8	1.7	105.6	1133.7	12.5	52.3	1700.6	18.8	32.6

Abbreviation: ICER: incremental cost-effectiveness ratio, DALY: disability adjusted life years. In Scenario 1, the DALY was assumed 1.13 with herd immunity level of 50% and basic doses of vaccines; in Scenario 2, we assumed DALY as 4, with herd immunity level of 50% and basic doses of vaccines. In Scenario 3, we considered adding a booster dose, with a DALY of 1.13 and a herd immunity level of 50%; in Scenario 4, we assumed DALY at 1.13, herd immunity level at 75%, and adding a booster dose.
